# Virtual first impressions: Zoom backgrounds affect judgements of trust and competence

**DOI:** 10.1371/journal.pone.0291444

**Published:** 2023-09-27

**Authors:** Abi Cook, Meg Thompson, Paddy Ross

**Affiliations:** Department of Psychology, Durham University, Durham, United Kingdom; University of Guelph, CANADA

## Abstract

Trait inferences from first impressions are drawn rapidly and spontaneously. However, the Covid-19 pandemic forced interactions online introducing differential influential factors on first impressions. As such, there is an absence of research investigating video background on videoconferencing impression formation. This study explored the influence of video background, facial expression, and gender on first impressions of trustworthiness and competence. Video background affected trustworthy and competence perceptions with Plants and Book backgrounds scoring highly on both dimensions while the Home and Novelty backgrounds consistently received the lowest ratings. Happy faces were perceived as more trustworthy and more competent while female faces were also rated as more trustworthy and more competent, regardless of the background they were using. The explanations for these findings are discussed, along with future directions for research and the implications for videoconferencing use.

## Introduction

First impressions are formed instantly. Within the first few seconds of meeting someone, we spontaneously draw inferences about their character traits [1]. Social judgements of trustworthiness, competence, likability, aggressiveness, and attractiveness are made in a few milliseconds [2]. Despite some errors in trait inference [3], immediate first impressions are remarkably robust [4]. They may not always be accurate, but often people share a consensus on the impression [3, 5], suggesting a shared social experience [6]. First impressions develop at an early age [7] and consensus of trait evaluations are shared cross-culturally [8]. This suggests an adaptive function of first impressions to determine others’ intentions, as friends or foe, and their agentic capacity to act on intentions [9]. The Stereotype Content Model (SCM) proposes interpersonal judgements are captured along these dimensions of warmth (trustworthiness, friendliness) and competence (capability, assertiveness) [10]. This parallels the trustworthiness and dominance dimensions identified from analysis of 13 social traits spontaneously judged from faces [11]. Trustworthiness and competence are therefore important dimensions for social judgements. First impressions influence significant real-world outcomes, including electoral success [12], financial success [13], criminal sentencing decisions [14] and mating choices [15]. These outcomes have driven extensive research into the influencing factors on first impressions [2].

However, the way we meet people is changing. The COVID-19 pandemic forced many of our interactions online through videoconferencing when face-to-face meetings were restricted [16]. Videoconferencing enables live audio and video communication. Platforms including Zoom and Microsoft Teams saw dramatic user increases from 10 million to 300 million and 20 million to 75 million respectively from late 2019 to April 2020 [17, 18]. The platforms facilitate remote working in organisations, businesses, and education, as well as enabling social connections [19, 20]. Within these contexts, strangers meet virtually for the first time, including in video interviews [21] and virtual first dates [22], demonstrating the relevance and necessity of investigating first impression formation in videoconferencing interactions. Virtual meetings are evolving into a permanent feature of the professional environment because they are convenient, efficient, and cost-effective. Predications estimate 75% of business meetings will occur through videoconferencing by 2024 [23], a prediction that is on target as Microsoft Teams had 270 million users in 2022, a 260% increase from the height of global lockdowns in April 2020. This clearly demonstrates the lasting presence of videoconferencing in a post pandemic world, necessitating a line of research that explores first impressions in a videoconferencing context.

Videoconferencing platforms simulate face-to-face meetings yet there are distinct situational differences between in-person and virtual interactions. In face-to-face meetings, there is limited control of the environment. In contrast, individuals can change the videoconferencing environment by blurring their background or selecting a virtual background. This disparity is significant because visual context influences first impressions. Catergorisation of facial expression was faster and more accurate when viewing a congruent emotional background [24]. Similarly, positive and negative contexts induce positive and negative evaluations respectively, regardless of facial expression [25]. In addition to affective contexts, threatening visual backgrounds influence evaluations. Neutral faces are judged as more dominant when a downward pointing triangle, known to convey threat, is in the background [26] while only threatening backgrounds (e.g. a room with blood on the floor) influenced trustworthiness judgements compared with neutral backgrounds (e.g. a cornfield) [27]. Further, Mattavelli (2022, 2023) has shown that the extent to which trust judgements by these contexts depends on the face-context relationship [28, 29]. For instance, a human-related threatening object (e.g. a bloody knife) was found to be more effective at influencing trust than a non-human related threat (e.g. a tornado). The target in these contexts was seen to have a role in these contexts (as a victim) and this role influenced the trait attribution. The role of context in facial processing is further supported by the effect of emotional background scenes on event-related potentials [30]. These findings indicate that first impressions are significantly influenced by contextual visual factors, likely through a top-down, adapted mechanism, therefore video background could differentially impact first impression evaluations compared to in-person meetings. The present study seeks to investigate the influence of visual context, signalled by video background, on evaluations of trustworthiness and competence.

Videoconferencing platforms provide the opportunity to alter virtual environments. Individuals may present a certain image of themselves to influence character perceptions, such as tidying up or blurring background mess to appear organised. This is particularly likely in interactions with strangers where there are no predisposed impressions. Presentation is an important part of impression formation as formally dressed job applicants are rated as more competent and have dramatically increased hiring recommendations compared to informally dressed candidates (97% vs. 14%) [31]. Virtual background could be considered an extension of professional appearance because personal spaces evoke character inferences [32, 33]. Survey research of professionals supports this notion as video background evaluations parallel dress attire, with background judged to be more important than clothing choice [34]. Virtual backgrounds have therefore been characterised as the new business suit [35]. However, the professionality of different video backgrounds has been debated. Viewing other’s homes in a work context is deemed unprofessional [35], supported by empirical research that found video interviews recorded from a bedroom were perceived as less professional than a home office environment [36]. Similarly, recruiters perceived job applicants to be unprofessional when they interviewed “in front of inappropriate backgrounds, photos or posters” [37]. Research points to the conclusions that viewing a personal space is unprofessional and may lead to negative evaluations on competence judgements. Experts have therefore advocated for the use of blank walls as video backgrounds [21, 38]. However, there exists a conflict between professionality and authenticity. Compared with a blank wall or virtual background, viewing the speakers’ actual room led to higher impressions of trustworthiness (73%), authenticity (65%), expertise (52%) [34] leading to some experts recommending using objects to frame positive evaluations [39]. This compromise parallels social research that has noted a compensation effect between judgements of competence and trustworthiness [40, 41]. Video background is clearly utilised to make personality attributions. Yet presenting oneself as professional and competent, by using blank walls and blurring backgrounds to hide personal space, may compromise perceptions of authenticity and trustworthiness. The present study aims to resolve this debate and determine if video background compromises competence and trustworthiness evaluations.

Despite the increase of videoconferencing, there is limited research investigating the influence of video background on first impression formation. Contrary to expectations, the sparse literature has indicated a diminished role for video background, with professional, personal, and blurred backgrounds not influencing initial impressions and final interview outcomes despite a relationship between final interview scores and initial impressions [36, 42]. This research demonstrates the importance of making a good first impression in video contexts but suggests video background is not an influencing factor. However, confounds in object type included within the backgrounds may explain these results. Research distinguishes between functional and symbolic objects where functional objects are practical and promote activity, associated with competence, and symbolic objects hold emotional significance, associated with warmth [43]. In Scott (2022), the professional background contained both functional (books) and symbolic objects (plants, diploma) which may have affected the findings. However, similar null results of video background were reported for big 5 personality trait inferences and hiring recommendations, even when backgrounds separated functional and symbolic objects [42]. The null findings could be explained by the studies being conducted in video interview contexts. While efforts were made to control for verbal cues such as pitch and tone of voice, and non-verbal cues such as gestures, gaze and facial expressions, there may have been differences that varied systematically with the background and subsequently confounded the results. The present study will use static photos instead of videos to truly capture the influence of background on initial first impressions without the influence of other verbal and non-verbal cues. This is particularly relevant for videoconferencing as platforms enable you to join muted with your camera on. Furthermore, the present study will expand on these studies by investigating the effect of background of evaluations of competence and trustworthiness which have both been found to be relevant to professional and videoconferencing contexts [44, 45]. The impact of video background on first impressions should not be discarded based on scarce literature thus more research is needed before drawing any conclusions. The primary objective of the current study is therefore to establish the influence of video background on first impressions of competence and trustworthiness.

Video background is a distinct non-verbal cue that extricates videoconferencing from in-person meetings. Virtual interactions are further distinguished through an absence of body language, identified as a significant influence in first impressions [46]. Even when camera frames allowed for body language, webcams framing the face from the shoulders up were preferred [47]. This indicates facial expressions are a principal non-verbal cue in videoconferencing. Facial expressions communicate valuable information about others’ feelings and intentions. The influence of facial expressions on first impressions has been well-established and derives from the adaptive benefit of reacting appropriately; approaching or avoiding those who look happy or angry [48]. It is therefore unsurprising that happy faces are perceived as high in trustworthiness while angry faces receive low trustworthiness ratings [49, 50]. The relationship is less clear for competency. Smiling predicts competence however this derived from a composite of non-verbal cues and physical attractiveness, obscuring the independent effect of smiling particularly considering the correlation between physical attractiveness and competency [51, 52]. However, research has found a negative relationship between smiling and competence judgements in expert and novel interactions [53]. For intelligence, a trait included in competence dimensions [54], there are also mixed results. Smiling individuals are perceived as more intelligent than the same non-smiling individuals [55, 56] while other studies found an opposite or null relationship [57, 58]. These effects are likely mediated by cultural differences and can partly be explained by stimuli variations. Smiling is thought to influence competency perceptions because smiling signals high self-confidence and self-esteem which typically result from high agency and competence [59]. The present study aims to resolve the literature debate on the influence of smiling on competency judgements and to evaluate the established influence of smiling on trustworthiness perceptions. Furthermore, contextual cues can compete with facial expressions in trait inferences during first impressions [60] therefore the interaction between facial expression and video background will be explored to determine if facial expression can override potential negative implications of background.

In addition to facial expressions, gender can exert a powerful influence on impression formation with a consistent positive correlation demonstrated between masculinity and perceived competence, and femininity with perceived warmth [61–63]. These perceptions prevail cross-culturally and could be derived from sexually dimorphic features [64]. Females possess more infantile features, including large foreheads and wide eyes which are judged as more trustworthy and less dominant [65]. In contrast, male face width, linked to testosterone levels, drives higher dominance and lower trustworthiness ratings [66, 67]. Higher competence ratings are further associated with masculine features and faces manipulated to appear competent were more likely to be classified as male [68]. Gender differences in first impressions within videoconferencing contexts are important to establish because gender biases exacerbate workplace inequality where videoconferencing is increasingly becoming the preferred method of communication [23]. Women are more likely to be discriminated against in a professional environment [69, 70] and are more likely to face obstacles when entering or staying in a field that requires intellectual competence [71, 72]. The present study will therefore investigate differences in trustworthiness and competency perceptions of male and female faces in a videoconferencing context. It will further explore the interaction effect of gender with video background offering a novel research area to the literature which has not previously been examined.

The present study will examine the effect of video background, facial expression, and gender on first impressions of trustworthiness and competence in a videoconferencing context. Participants viewed female and male faces with happy and neutral expressions overlaid onto six backgrounds to investigate these effects. The six virtual backgrounds selected consisted of a living room (referred to as ‘home’), a blurred version of the same living room (‘blurred home’), a bookcase, house plants, a blank wall and an entertaining, ‘novelty’ background. These include typical “working from home” videoconference backgrounds to investigate the potential compromise between competence and trustworthiness. Despite convincing evidence for the effect of context, the findings of video background research are inconclusive. It is therefore hypothesised that background will influence trustworthiness and competence evaluations, however no direction is predicted (Hypothesis 1). The evidence supporting a relationship between smiling and trustworthiness is compelling. While there is mixed support for the effect of smiling on competence, evidence indicates a positive relationship therefore it is hypothesised that happy faces will be viewed as more trustworthy and more competent than neutral faces (Hypothesis 2). Based on the literature of gender stereotypes, it is predicted that males will be judged as more competent, and females will be judged as more trustworthy (Hypothesis 3). The interaction effect of video background with both facial expressions and gender will also be explored.

## Method

### Participants

A power analysis using G*Power 3 [73] revealed that to detect an effect of *η*^*2*^_*p*_ = .01 with over 95% power in our main effect for Hypothesis 1, we would need at least 165 participants. 167 participants completed the study in July and August 2022 (115 females, 50 males, 2 non-binary) between the ages of 19 and 68 (*Mage* = 35.01, *SD* = 15.16). Participants were informed the study related to first impressions online when using video conferencing platforms. They were recruited through volunteer sampling from various sites. These included Prolific, a survey site where participants were paid £1.66 for completing the 10-minute survey, the researcher’s social networks via advertisements on social media and from an undergraduate sample at Durham University via a departmental advertisement. Those who were eligible received course credit for participating. All data was anonymised, and no identifiable information was collected. The study obtained ethical approval from the Department of Psychology at Durham University (PSYCH-2020-12-10T09_02_46-qwpf21) and all participants gave informed written consent.

### Design and measures

A within participants design was used with three independent variables, background with 6 levels: ‘Home’, Blurred Home’, ‘Bookcase’, ‘Plants’, ‘Blank’ and ‘Novelty’; gender of facial stimuli with 2 levels: Male and Female; and facial expression with 2 levels: Happy and Neutral. First impressions were measured by the two dependent variables, evaluations of trustworthiness and competence. After viewing each stimulus, participants responded to two items “How trustworthy is this individual?” and “How competent is this individual?” which were both rated on a 7-point Likert scale. For the trustworthy dimension this ranged from 1 = “*Very Untrustworthy”* to 7 = “*Very Trustworthy”* and similarly the competence dimension ranged from 1 = “*Very Incompetent”* to 7 = “*Very Competent*.*”*

### Stimuli

Stimuli comprised of male and female faces with happy and neutral expressions superimposed onto one of 6 virtual backgrounds, framed within a Zoom border to simulate the experience of a videoconference call. 36 Caucasian faces (18 female, 18 male) displaying both happy and neutral expressions were obtained from the Radboud database [74], comprising 72 images in total. For each background, 3 males and 3 females with both happy and neutral expressions were randomly overlaid such that there were 12 faces for each background. The face-background combinations were randomised for each participant. Happy and neutral expressions were selected because they replicate the common facial expressions in videoconference contexts. Participants rated all 72 stimuli therefore viewed each face twice (once with a neutral expression and one with a happy expression). While this is not strictly a first impression, individuals are often unable to recognise the same unfamiliar individuals from different photos [75]. In the present study, participants were not identifying faces and response time averaged 9.2 seconds for each face therefore it is highly unlikely that viewing each face twice will have affected the results.

**Fig 1:**
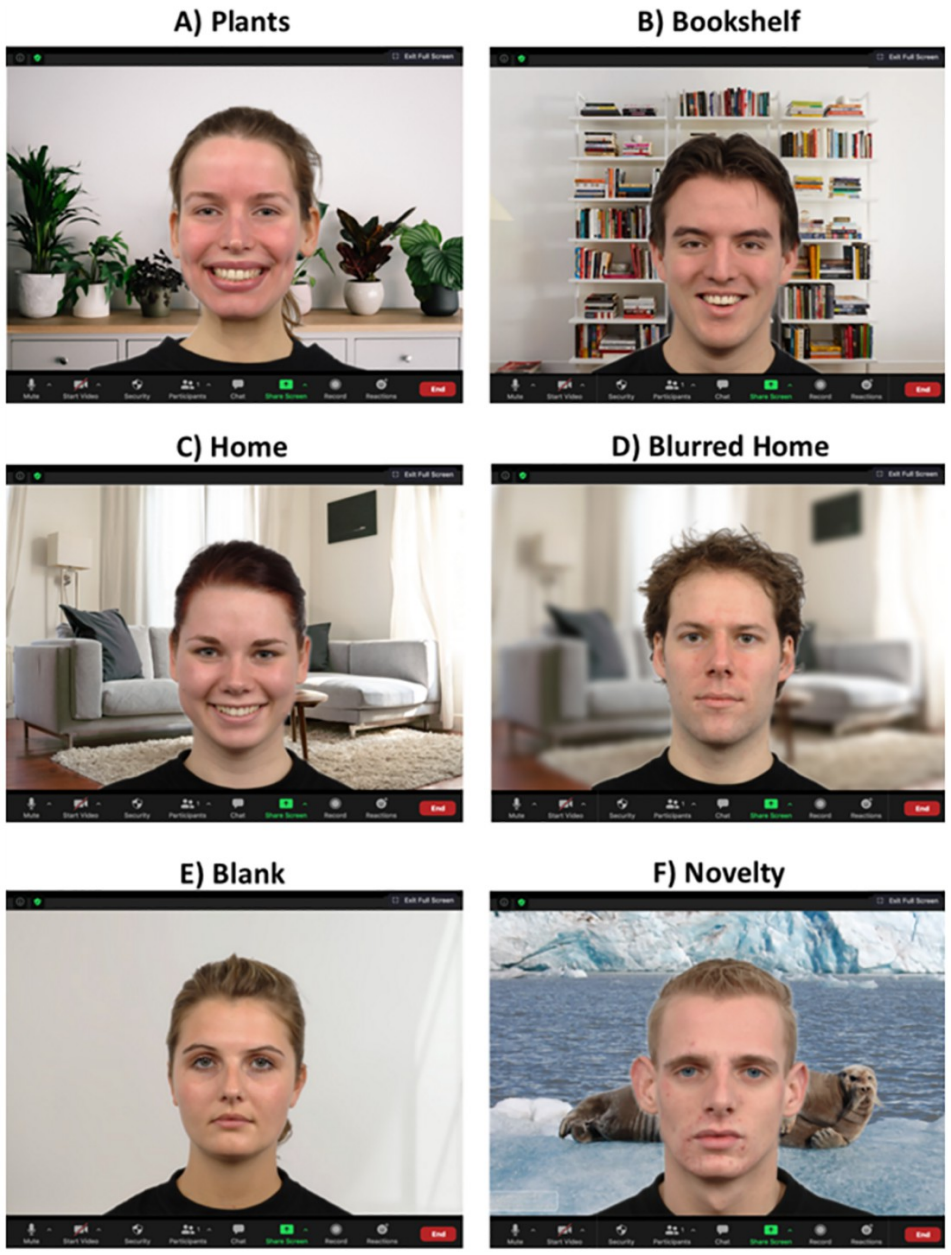
Sample Stimuli: A) Happy female on plants background B) Happy male on bookcase background C) Happy female on home background D) Neutral male on blurred home background E Neutral female on blank background F) Neutral male on novelty background.

Six virtual backgrounds were selected consisting of Home, Blurred Home, Bookcase, Plants, Blank and Novelty. The background images were obtained from public domain websites because the virtual backgrounds offered by videoconferencing platforms, including Zoom and Microsoft, were not in comparable resolution, perspective, colour scheme or lighting which are conditions found to impact perceptions [76, 77]. Examples of stimuli are shown in Fig 1.

### Procedure

Data was collected through an online questionnaire using the Qualtrics survey platform. Participants read the information sheet followed by a consent form where informed consent was given. Participants were presented with a face overlaid onto one of the 6 backgrounds framed with a Zoom border to simulate videoconferencing. After viewing each stimulus, participants responded to two items “How trustworthy is this individual?” and “How competent is this individual?” which were both rated on a 7-point Likert scale. Participants viewed all 72 stimuli, randomised by Qualtrics. There were no time restrictions for responses because additional time exposure does not alter first impressions which form in under a second [2]. Furthermore, unrestricted time better represents a real videoconference scenario. Those who successfully completed the survey were debriefed reimbursed (if relevant, dependent on recruitment) and thanked for taking part in the study. Participants took approximately 10 minutes to complete the experiment.

## Results

Two repeated measures ANOVA were conducted to determine the effect of background, gender, and facial expression on first impressions of trustworthiness and competence. Where Mauchly’s test of sphericity was violated, degrees of freedom were corrected using Greenhouse Geisser estimates for ε<0.75 and Huynh-Feldt estimates for ε>0.75. The results for trustworthiness are presented first, followed by the results for competence. All post-hoc comparisons are Bonferroni corrected.

### First Impressions of trustworthiness

#### Background

**Fig 2:**
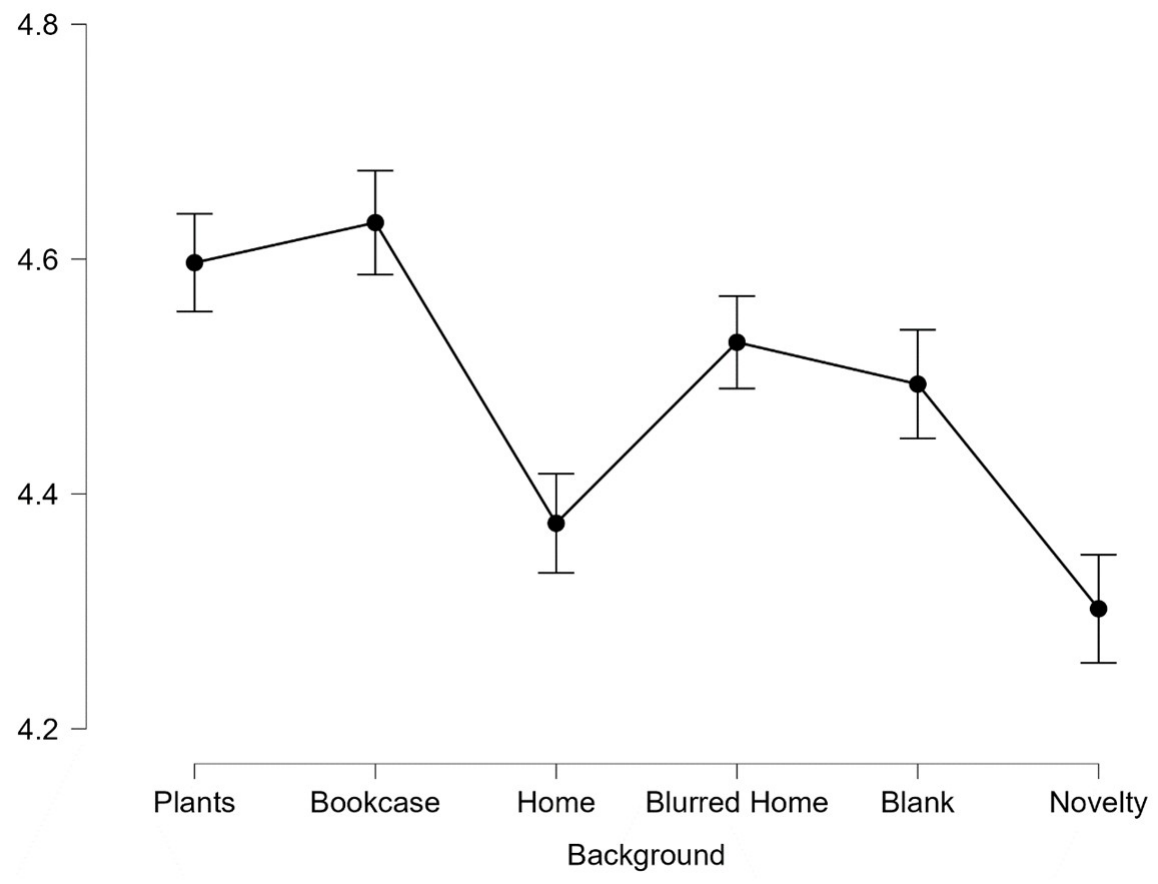
Average scores for trustworthiness across all 6 backgrounds. Error bars represent SEM.

First impressions of trustworthiness differed significantly across backgrounds, *F*(4.46 735.33) = 23.31, *p* < .001, *η*^*2*^_*p*_ =. 124. Mauchly’s test indicated that the assumption of sphericity had been violated, χ2(14) = 52.86 *p* < .001, therefore degrees of freedom were corrected using Huynh Feldt (ε = .85). Descriptive statistics are presented in Fig 2.

Post-hoc pairwise comparisons revealed faces with a background of Home (*M* = 4.38, *SD* = .22) and Novelty (*M* = 4.30, *SD* = .23) were perceived as being significantly less trustworthy than those with backgrounds of Plants (*M* = 4.60, *SD* = .23), Bookcase (*M* = 4.63, *SD* = .24), Blurred Home (*M* = 4.53, *SD* = .23) and Blank (*M* = 4.49, *SD* = .23), all *p* < .001. Blurred Home was additionally more trustworthy than Home (*p* < .001) and Novelty (both *p* = .05). However, there was no significant difference between the highest rated backgrounds (Plants and Bookcase) *p*>.99 or between the lowest rated backgrounds (Home and Novelty), *p*>.99.

#### Facial expression

For facial expressions, happy faces (*M* = 5.17, *SD* = .23) were perceived as significantly more trustworthy than neutral faces (*M* = 3.80, *SD* = .24), *F*(1,165) = 511.0, *p* < .001, *η*^*2*^_*p*_ = .756.

There was also significant interaction effect between facial expression and background on first impressions of trustworthiness *F*(4.9, 808.459) = 18.31, *p* < .001, *η*^*2*^_*p*_ = .1.

**Fig 3:**
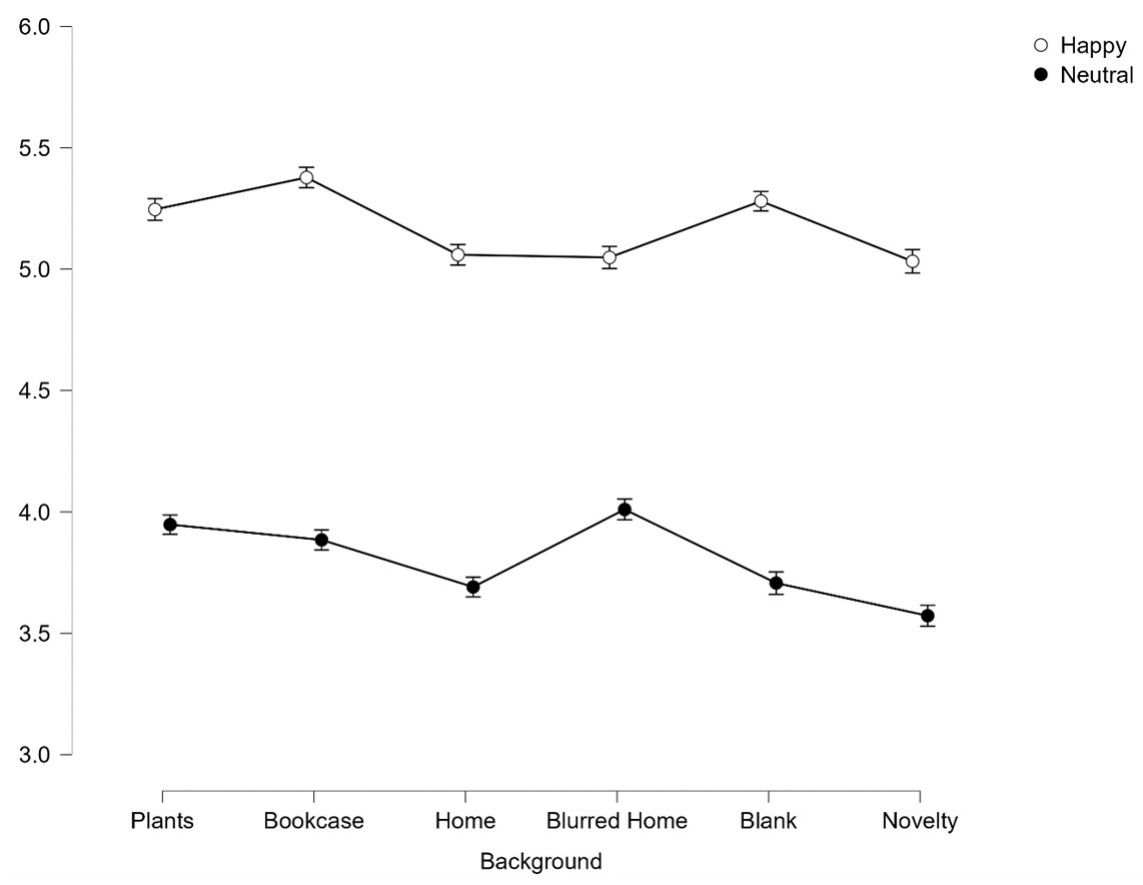
Average scores for trustworthiness across all 6 backgrounds split by facial expression. Error bars represent SEM.

Despite this significant interaction, post-hoc pairwise comparisons reveal that there is an additive effect of facial expression, with happy faces being perceived as significantly more trustworthy than neutral across all backgrounds (all *p* < .001, see Fig 3).

#### Gender

There was a significant main effect of gender *F*(1,165) = 140.37, *p* < .001, *η*^*2*^_*p*_ = .460. Female faces (*M* = 4.69, *SD* = .21) were rated as significantly more trustworthy than males (*M* = 4.29, *SD* = .23), *p* < .001.

There was also a significant interaction effect between gender and background on perceptions of trustworthiness, *F*(4.98, 821.68) = 15.64, *p* < .001, *η*^*2*^_*p*_ = .087.

**Fig 4:**
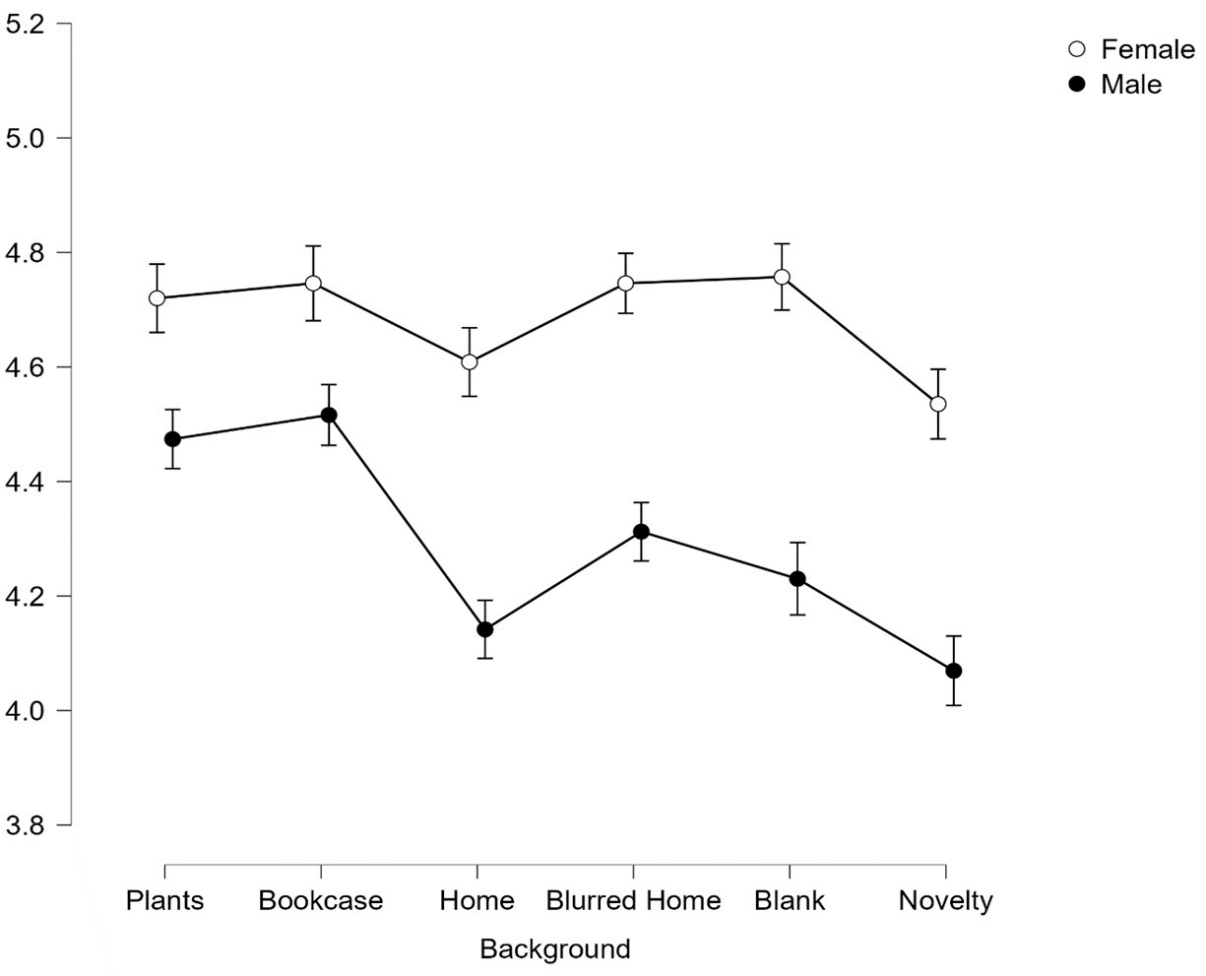
Average scores for trustworthiness across all 6 backgrounds split by gender. Error bars represent SEM.

To explore the interaction effect, post-hoc pairwise comparisons reveal that similar to Facial Expression, Gender produced an additive effect on perceived trustworthiness in the backgrounds with Females judged to be significantly more trustworthy than males across all backgrounds (all *p <* .001, see Fig 4). Interestingly for the Female faces, the Home background was no longer found to be significantly less trustworthy (*p*>.99) than the top two backgrounds of Plants and Bookcase. This overall effect therefore appears to be driven by the male faces in a Home background.

We also found a significant 3-way interaction between Emotion, Gender and Background, *F*(5, 825) = 15.64, *p* < .001, *η*^*2*^_*p*_ = .087.

*Happy*. Fig 5.

**Fig 5:**
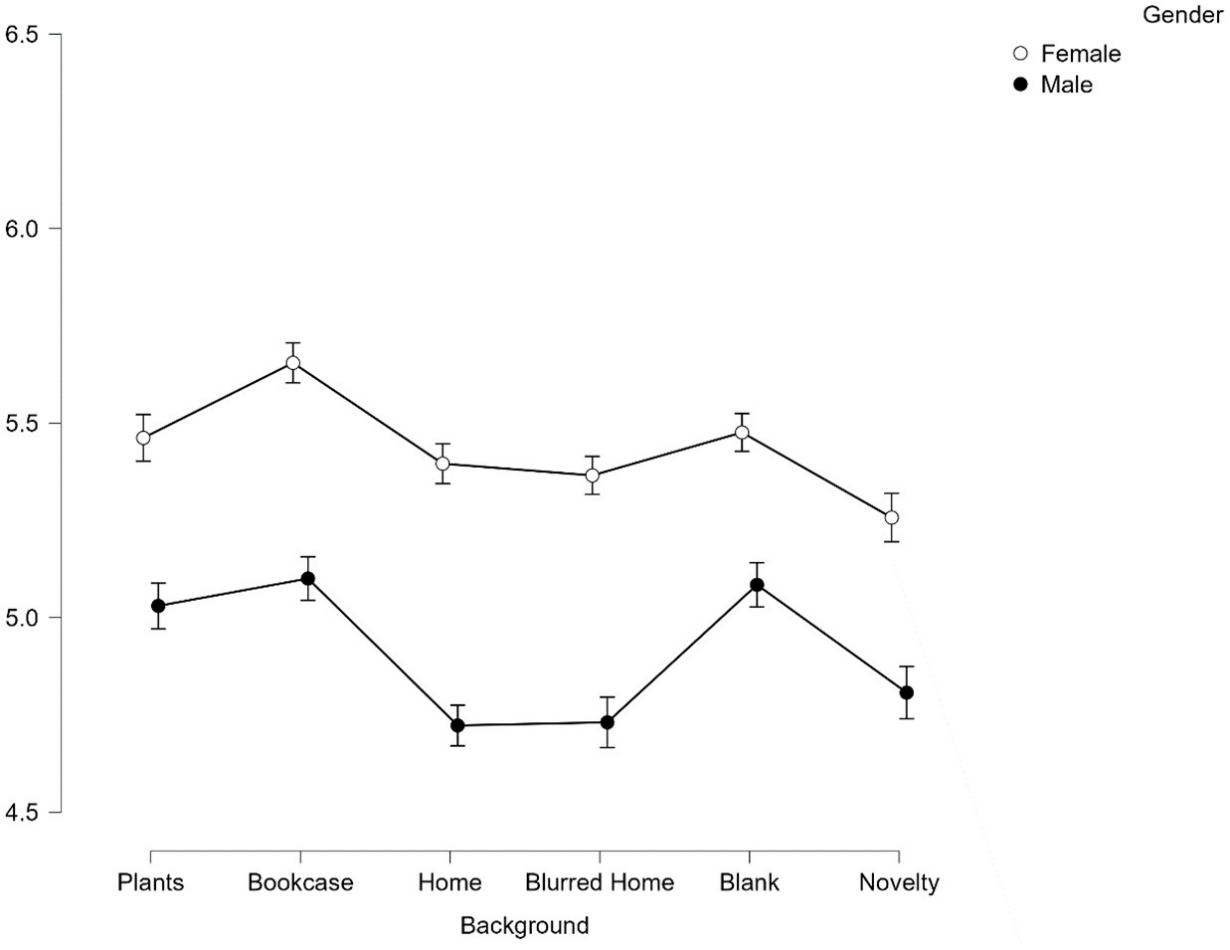
Average scores for trustworthiness from happy faces across all 6 backgrounds split by gender.

*Neutral*. Fig 6.

**Fig 6:**
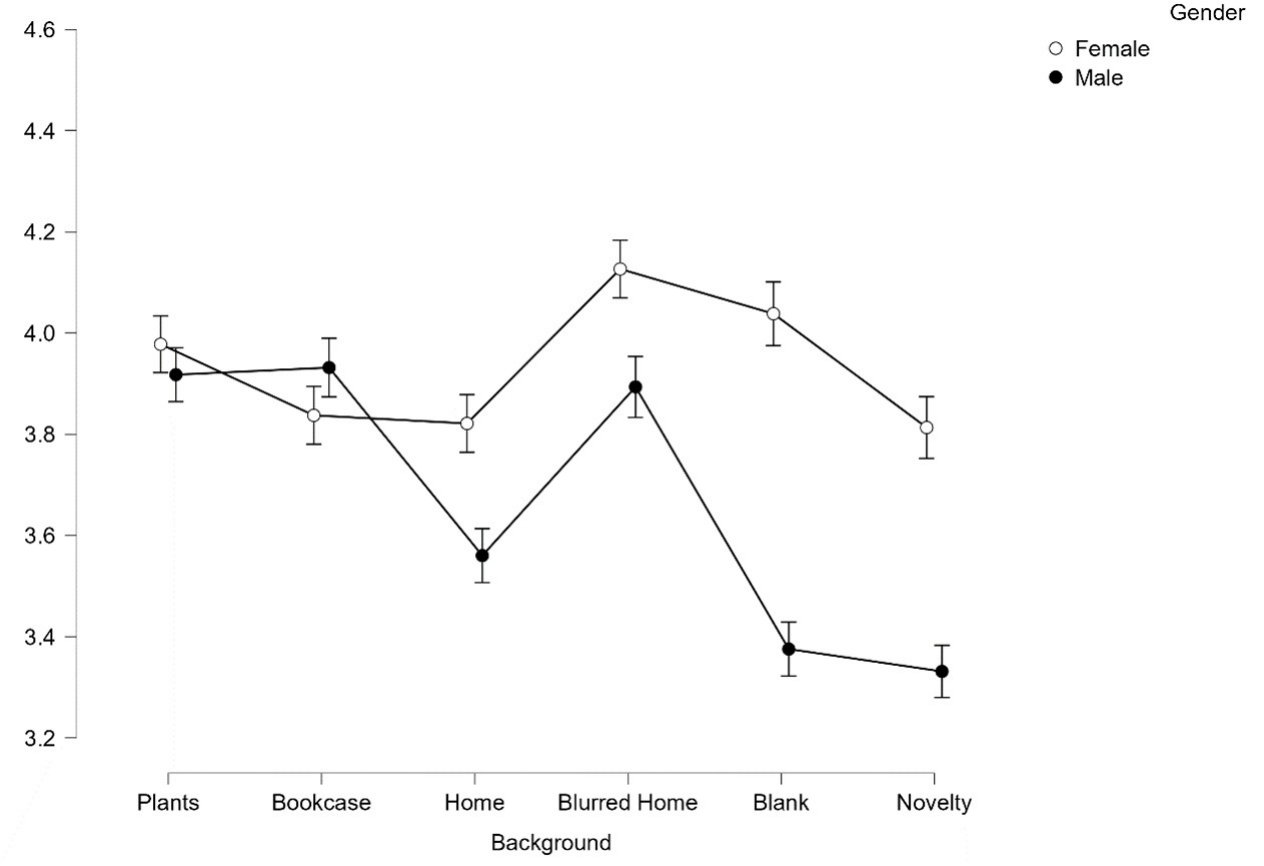
Average scores for trustworthiness from neutral faces across all 6 backgrounds split by gender.

When split by emotion (see Figs 5 and 6), we find that Females are judged as significantly more trustworthy than Males in every background when they are portraying happiness (all *p* < .001). However, when portraying a neutral expression, Females are judged to be more trustworthy than Males only in Home (*p* < .05), Blank (*p* < .001) and Novelty (*p* < .001).

### First impressions of competence

#### Background

On evaluations of competence, first impressions differed significantly between backgrounds, *F*(3.29,539.23) = 53.82, *p* < .001, *η*^*2*^_*p*_ = .247. Mauchly’s test of sphericity indicated a violation of sphericity (χ2(14) = 177.703, *p* < .001), therefore degrees of freedom were corrected using Greenhouse Giesser estimates of sphericity (*ε* = .66).

**Fig 7:**
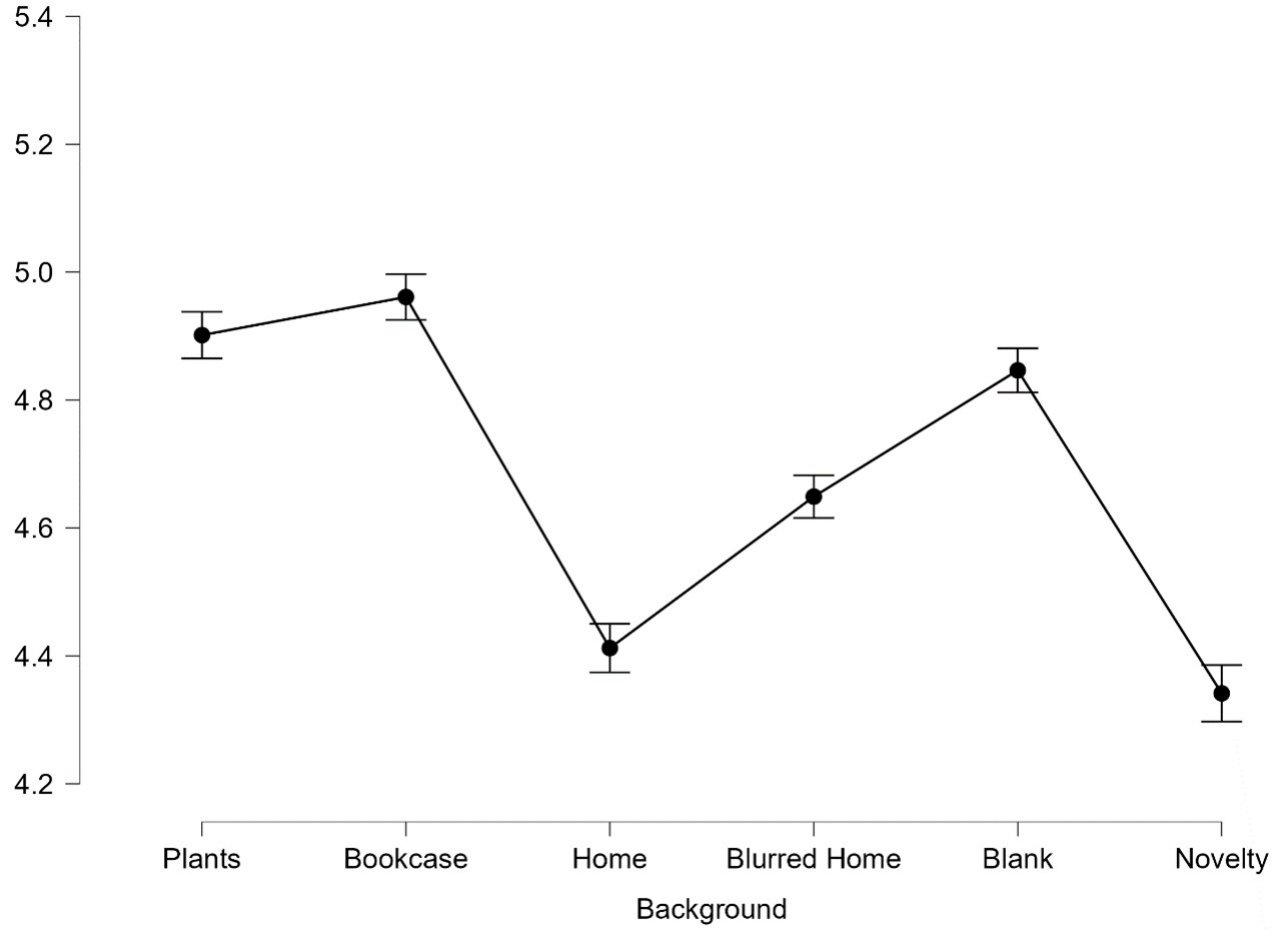
Average scores for competence across all 6 backgrounds. Error bars represent SEM.

Post-hoc pairwise comparisons revealed Bookcase (*M* = 4.96, *SD* = .24), Plants (*M* = 4.90, *SD* = .24) and Blank (M = 4.85, *SD* = .24) led to people being judged as significantly more competent than Home (*M* = 4.41, *SD* = .24), Blurred Home (*M* = 4.65, *SD* = .24) and Novelty (*M* = 4.34, *SD* = .24), all *p* < .001. There was no significant difference between the three highest rated backgrounds: Plants, Bookcase and Blank, *p*>.99 (see Fig 7).

#### Facial expression

**Fig 8:**
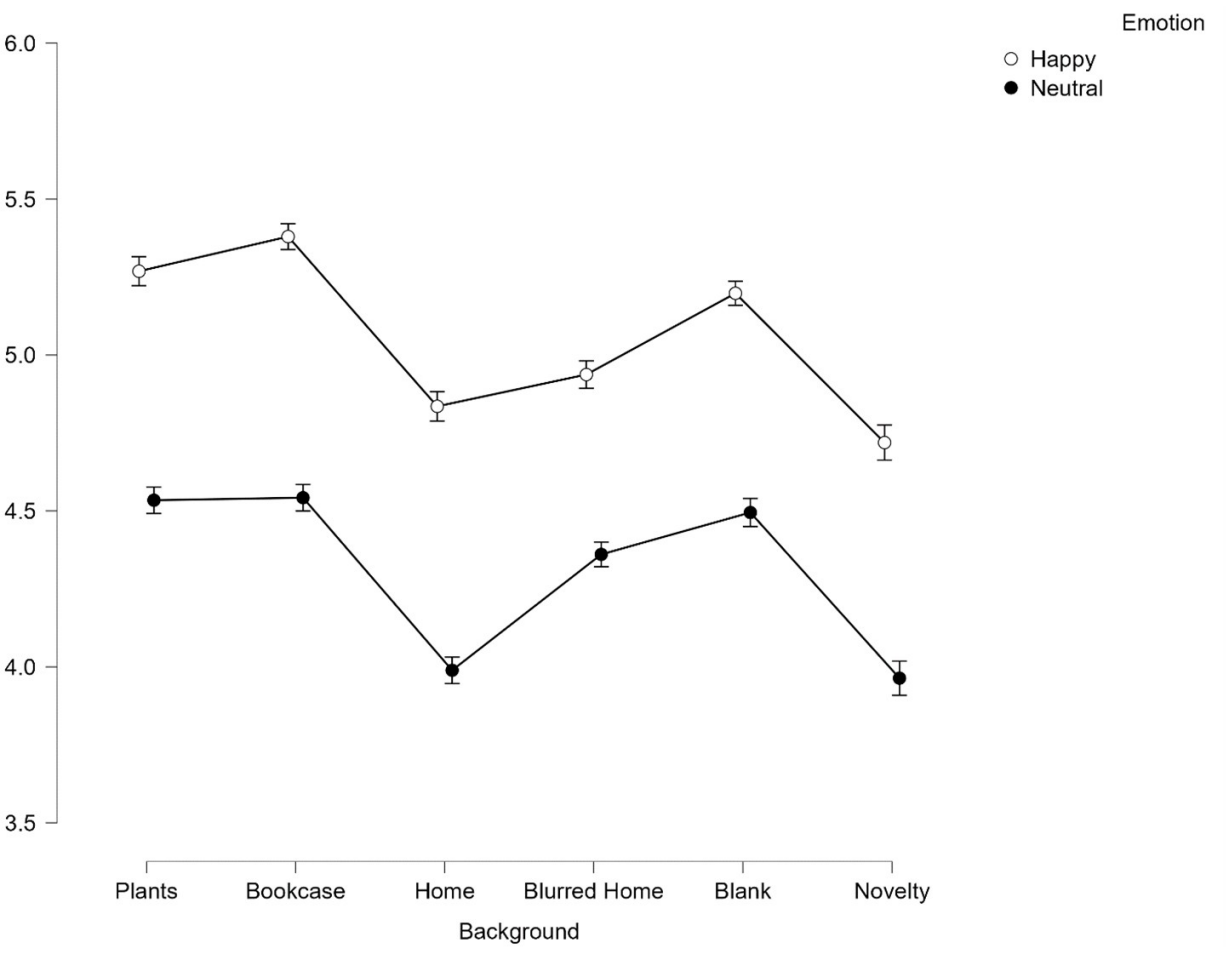
Average scores for competence across all 6 backgrounds split by emotion. Error bars represent SEM.

A significant main effect of facial expression was found for judgements of competency, *F*(1,165) = 154.16, *p* < .001, *η*^*2*^_*p*_ = .485. Happy faces (*M* = 5.06, *SD* = .023) were significantly more competent than neutral faces (*M* = 4.31, *SD* = .24), *p* < .001 (see Fig 8).

There was a significant interaction effect between facial expression and background *on competence* ratings, *F*(5, 825) = 5.58, *p* < .001, *η*^*2*^_*p*_ = .033. To explore the interaction effect further, Bonferroni corrected paired t-tests were conducted between happy and neutral faces at the 6 background levels. Happy faces were found to be judged as significantly more competent than neutral faces across all backgrounds (all p < .001).

#### Gender

**Fig 9:**
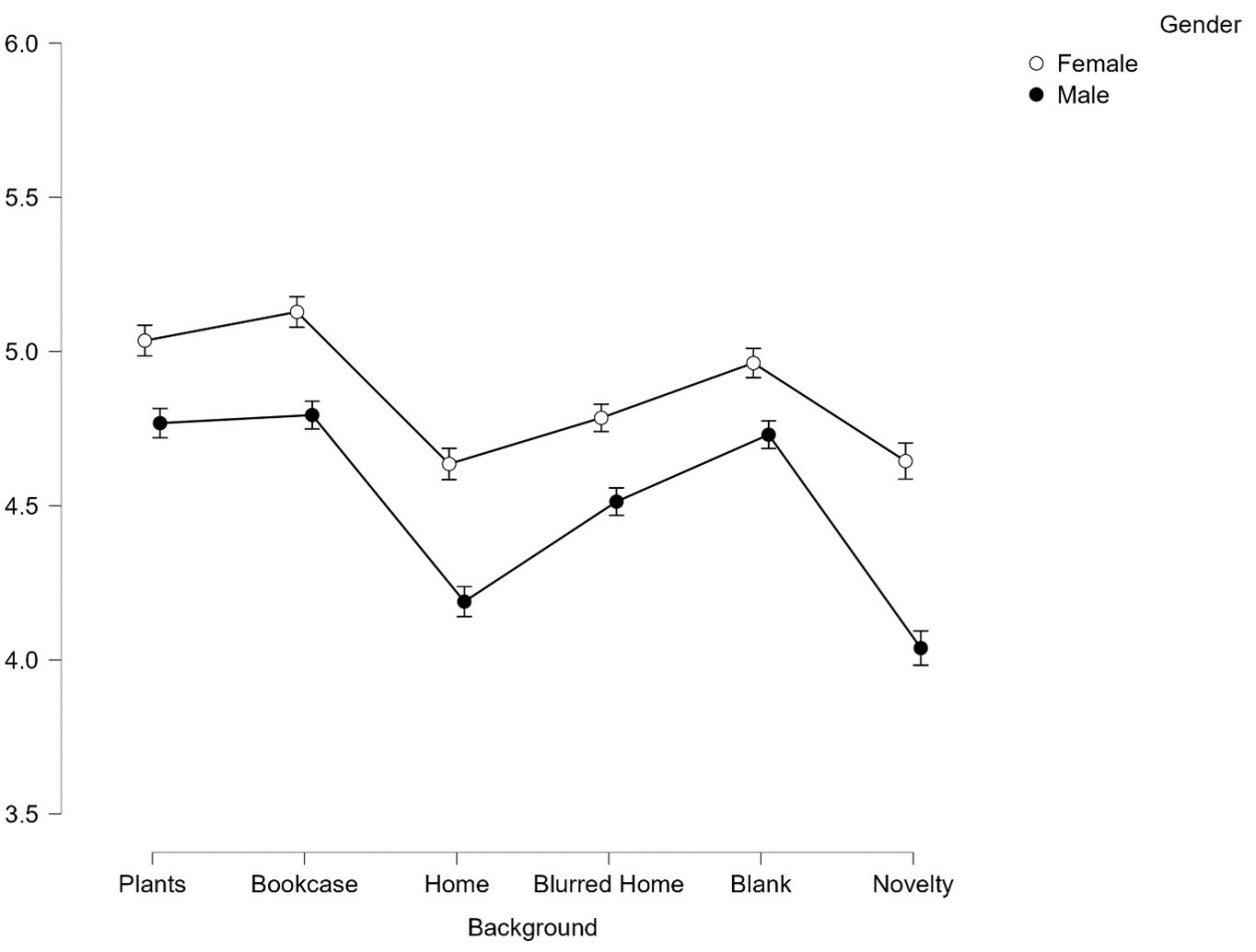
Average scores for competence across all 6 backgrounds split by gender. Error bars represent SEM.

**On evaluations of competence, female faces (***M* **= 4.87,** *SD* **= .22) were perceived as significantly more competent than male faces (***M* **= 4.51,** *SD* **= .22),** *F***(1,165) = 121.9,** *p* **< .001,** *η*^*2*^_*p*_ **= .426 (See Fig 9).**

There was also significant interaction effect between background and gender on competence perceptions, *F*(5,825) = 8.67, *p* < .001, *η*^*2*^_*p*_ = .05. Again post-hoc Bonferroni corrected paired t-tests revealed that Female faces were judged to be more competent than male faces across backgrounds (all *p* < .001 except for Blank background which was *p* < .005).

Finally, we found a significant 3-way interaction between Emotion, Gender and Background, *F*(5, 825) = 6.92, *p* < .001, *η*^*2*^_*p*_ = .04.

*Happy*. Fig 10.

**Fig 10:**
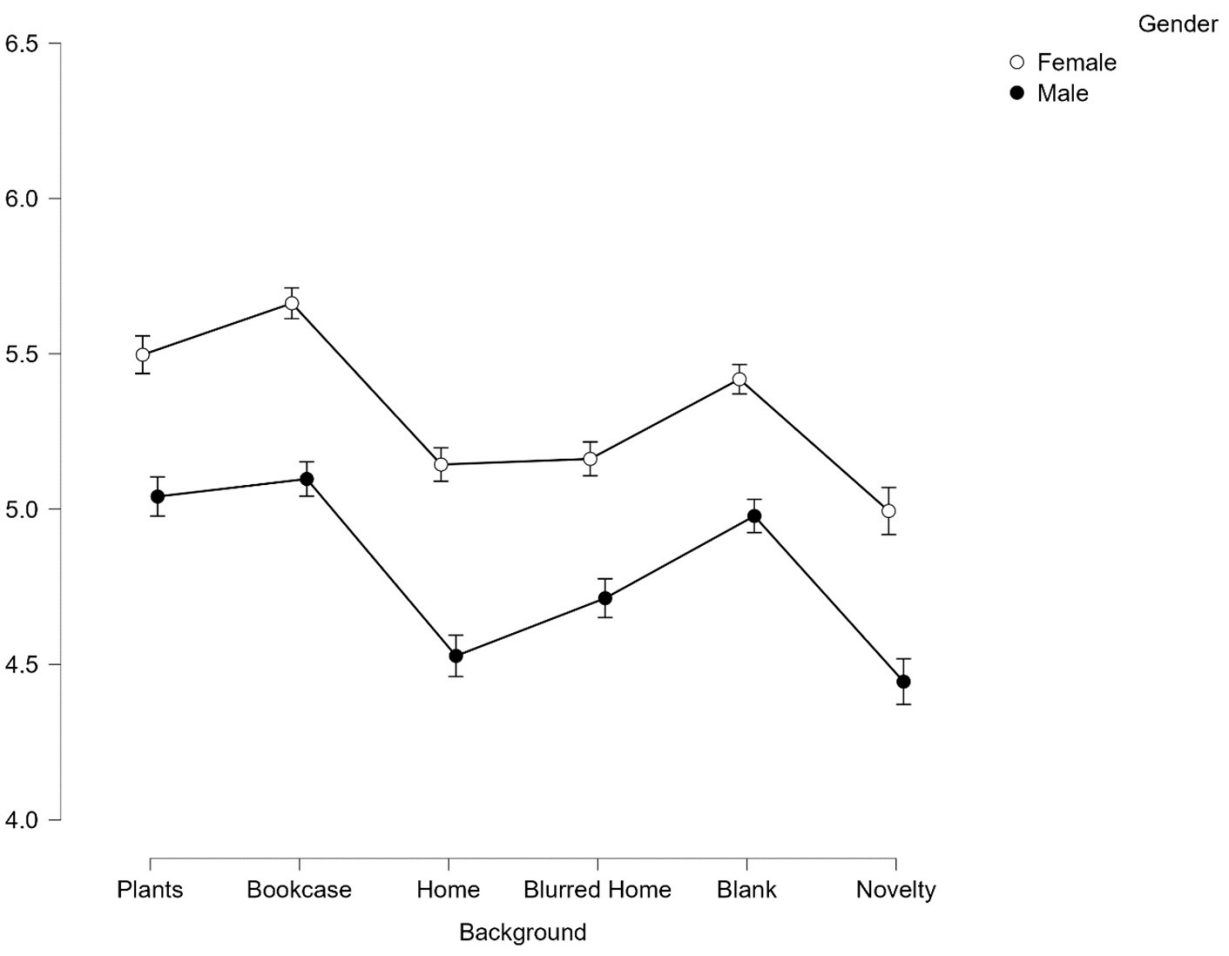
Average scores for competence from happy faces across all 6 backgrounds split by gender. Error bars represent SEM.

*Neutral*. Fig 11.

**Fig 11:**
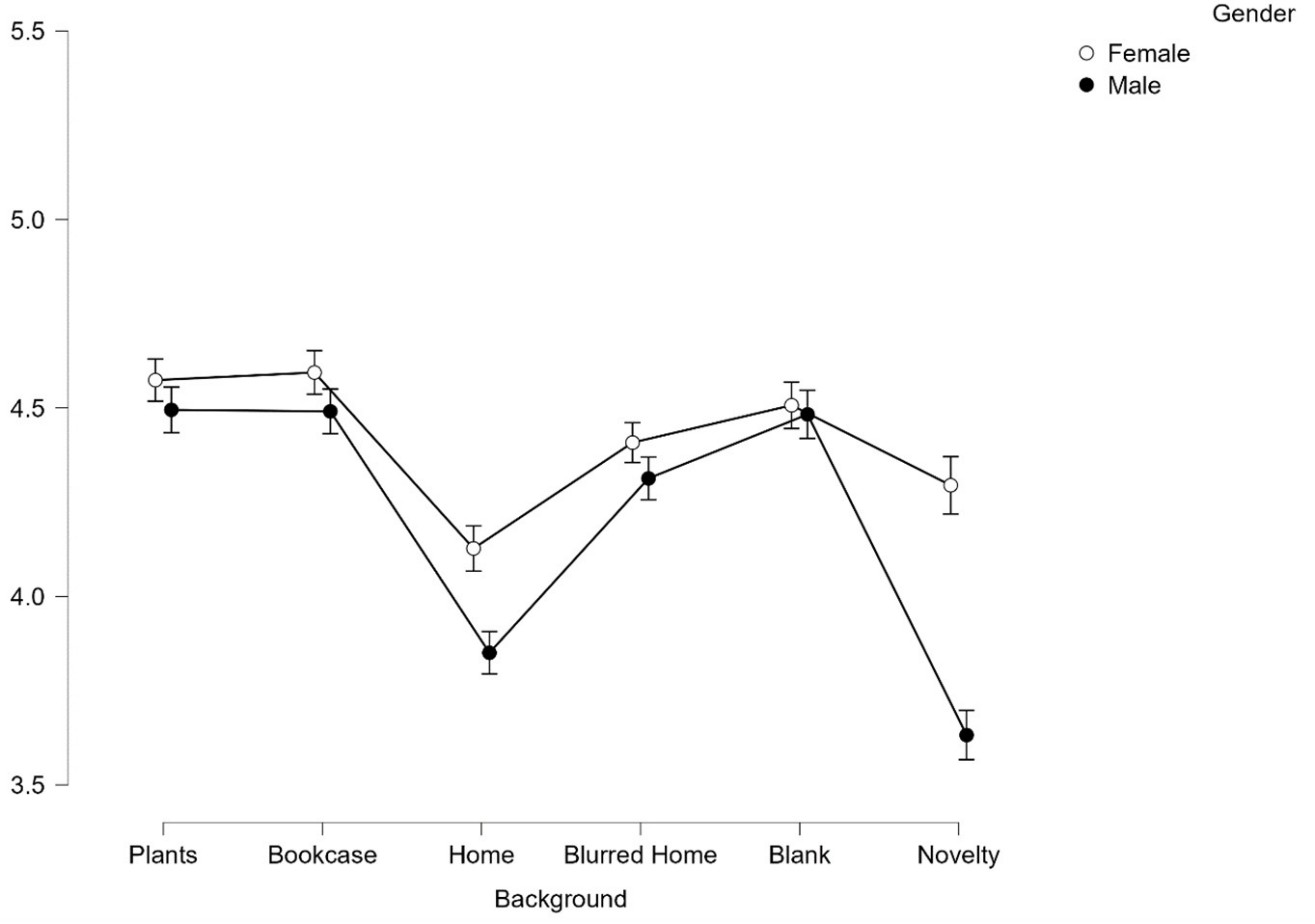
Average scores for competence from neutral faces across all 6 backgrounds split by gender. Error bars represent SEM.

When split by emotion (see Figs 10 and 11), we find that Females are judged as significantly more competent than Males in every background when they are portraying happiness (all *p* < .001). However, when portraying a neutral expression, Females are judged to be more competent than Males only in Home (*p* < .05) and Novelty (*p* < .001).

## Discussion

The present study investigated the influence of video background, facial expression, and gender on first impressions of trustworthiness and competence in a videoconferencing context. An effect of video background was found, supporting the first hypothesis. Faces presented on the plants and bookcase background were consistently rated as the most trustworthy and most competent, contrasting the home and novelty backgrounds which received lower trustworthiness and competence ratings. Facial expression influenced evaluations, consistent with the second hypothesis, with Happy faces judged as more trustworthy and more competent than neutral faces. The third hypothesis was partly supported. Females were perceived as more trustworthy, but they were also rated more competent, contrasting predictions of a male competence bias. The study found significant interaction effects of video background with both facial expressions and gender.

The effect of video background supports the first hypothesis, extending previous research on the influence of visual cues on first impressions into a videoconferencing context, demonstrating emotional or threatening contexts are not necessary conditions to affect trait evaluations [24, 27]. For competence perceptions, the low ratings for faces in home and novelty backgrounds are consistent with the literature where virtual scenic backgrounds received low expertise ratings while backgrounds showing personal spaces are deemed unprofessional [34, 36]. The extension of professional appearance to include video background may explain these findings. Formally dressed individuals are rated as more competent because professional attire communicates maturity, capability, and success [31]. The home and novelty backgrounds could therefore be compared to informal attire because the backgrounds indicate a casual and relaxed attitude, suggesting the individual may not be conscientious and thus explaining evaluations of low competence. Faces in home and novelty backgrounds were also rated as low on trustworthiness which is somewhat surprising. For the novelty background, conventional wisdom suggests a person would be perceived as light-hearted and humorous. Perhaps as a virtual background, participants judged it to be hiding their personal environment, inducing perceptions of secrecy and dishonesty. This does not explain why faces on the blurred home background received relatively high trustworthiness ratings. However, faces on the home background were perceived as even less trustworthy than faces on the blurred home which cannot be explained by a concealment account because displaying your home exposes vulnerability. The finding is surprising because viewing the actual room of videoconference speakers increased perceptions of trustworthiness and authenticity in previous work, therefore one might have expected faces on the home background to have higher ratings [34]. The low ratings of faces on home and novelty backgrounds on both dimensions suggest there is an overall negative effect of these backgrounds. A general detrimental effect is supported by faces on the blank background being rated among the most competent, despite limited signalling of positive behavioural cues. This effect is likely induced by the negative implications of informality and unprofessionalism. The salience of competency judgements in videoconferencing contexts may drive this effect, particularly as participants judged both dimensions simultaneously. Negative competence evaluations may therefore have influenced trustworthiness evaluations or vice-versa. Future research should disentangle the dimensions by assessing trait inferences separately to determine if the findings are explained by an influence of competency judgements on trustworthiness perceptions or if a general negative effect is upheld.

The literature indicated a potential compromise between competence and trustworthiness, bolstered by findings of a compensation effect between warmth and competence [78]. However, the current study finds no evidence to support this relationship. Furthermore, the study failed to support a relationship between the functionality objects with competence. Faces on the bookcase background consistently received high ratings for both dimensions while faces on the home received the lowest ratings, yet both backgrounds included functional objects. Books were likely rated highly due to the positive relationship between literacy with intelligence and reading with academic performance [79, 80] which reflects the decisive preference for a wall with books or bookshelves in videoconferencing [34]. Faces on the plants background also scored highly on competence and trustworthiness perceptions which complements the established human preference for natural environments and the positive evaluations induced by house plants [81, 82]. The findings suggest objects do influence trait evaluation, but it is the qualities of the objects themselves and what they indicate about individuals’ character, and not their functionality or symbolism, that affects judgements. This supports the work of Matavelli (2022, 2023), as there appears to be a face-context relationship established by the perceiver (i.e. if the target is in front of books they might have read them all and be a smart person) [28, 29]. Further studies could explore this idea in more detail, perhaps by altering the type of book from the academic to novel or autobiography. If there is a face-context relationship that the perceiver is creating, then the quality of the reading material should matter as well as the quantity.

Consistent with the second hypothesis, facial expressions influenced first impressions. Happy faces were perceived as more trustworthy than neutral faces which confirms the well-established influence of smiling on trustworthiness evaluations [50, 83]. The adaptive value of facial expressions explains this finding. Smiling signals friendliness and prompts approach behaviour thus inducing perceptions of trustworthiness. Smiling further positively influenced competency judgements compared to neutral faces. A possible explanation is that smiling signals self-confidence, high-self-esteem, and success which typically result from high agency and competence [59]. The negative relationship found by previous research can be explained by culture differences in societal uncertainty where certainty expressed through smiling is be perceived as unintelligent [55, 84].

The present study did not manipulate the situational context, participants were only primed to imagine a virtual meeting. Competence evaluations can vary dependent on task and context with context mediating smiling perceptions [85]. Competence was negatively related to smiling in jobs that required a serious demeanour, perhaps because smiling was judged to be inappropriate for the job [86]. While the specific context was not manipulated, video background is a visual contextual cue which explains why an interaction effect between video background and facial expression was found. Smiling, associated with positive evaluations of trustworthiness and competence, appears to only reliably override the negative associations evoked by the home and novelty backgrounds in ratings of trustworthiness. Happy faces also enhanced the positive effect of books over the blank background for competence evaluations and over plants for trustworthiness. The present study enhances the literature by exploring this novel concept. However, an interesting avenue for future research would be to determine the influence of situational differences in videoconferencing interactions to assess the positive relationship found in the present study.

Regarding the third hypothesis, females were found to be more trustworthy and more competent than males which partly subverted expectations. The finding of females as more trustworthy reflects established findings supporting expectations of the communal female social role [87, 88]. However, contrary to hypothesis predictions, females were rated as more competent than males. These results may be explained by the attractiveness of feminine facial features which contribute to competence evaluations and ultimately mask gender biases. Oh (2019) found a masculinity bias for competence only when attractiveness was controlled for. The present study did not control for facial attractiveness thus a male competence bias may have been masked. Nevertheless, a lack of male competence bias may reflect changing gender stereotypes. A recent meta-analysis of gender stereotype public opinion polls demonstrated a profound social trend of increasing belief in gender competence equality, along with a decided female superiority among those who indicated a sex difference in competence [89]. Despite significant consensus among sex, education level, employment status, race, ethnicity, and generations, there was a small measure of female ingroup favouritism. The high proportion of female participants in the present study could explain the female competence advantage. Nevertheless, the present study’s findings are consistent with the literature, challenging the traditional gender stereotypes of male competence.

The study also found an interaction effect between gender and background. The convincing feminine superiority for trustworthiness appeared to refute the negative implications of the home and blurred home backgrounds such that the difference compared to the most trustworthy backgrounds was non-significant. Further, the feminine competency advantage increased the positive evaluations of competency for the bookcase background compared to blank. For males, however, the effect of background on trustworthiness and competency judgements was stable. For the public using videoconferencing, this has interesting implications. Men should be the most aware of their virtual background because of the stability of the video background effect. For females, video background is less important because while feminine advantages in both dimensions can counteract the negative implications induced by backgrounds.

The present study extends and improves upon the limited literature on virtual background by correcting for methodological limitations [36, 42]. Using static photos prevents confounds from verbal cues, including pitch and tone of voice, and non-verbal cues including gestures, gaze, and facial expressions, allowing investigation of the true influence of background. Furthermore, real faces were used which are comparable to the natural, unconstrained faces encountered in everyday life thus advancing previous research that used computer generated images and increasing the ecological validity of the present study. The use of facial stimuli with a direct, forward-facing gaze has been criticised for lacking ecological validity [90]. However, frontal gaze is particularly relevant for videoconferencing due to webcam positioning and features that allow individuals to join calls with their camera on, but audio muted. The facial stimuli used here therefore mimics actual initial videoconferencing interactions, facilitating an accurate and ecologically valid investigation of first impressions in videoconferencing; a clear strength of the present study.

One limitation of the current study is the variability of facial stimuli. Faces were also not pre-screened on traits such as attractiveness. Attempts were made to control for facial appearance using the maximum number of available faces to generate an average effect for each background. However, attractiveness influences first impressions of trustworthiness and competence [50, 68]. Similarly, backgrounds were carefully chosen based on equivalent resolution, lighting, and colour palettes because these variables influence trait evaluations [76, 77]. However, resource constraints made this feasible only to an extent. In addition, this study is limited by only using 6 backgrounds. In modern videoconferencing the background options are vast, with users even able to upload their own backgrounds. Future research should substantiate this study’s findings using pre-validated faces, many more examples of background categories and tailor-made backgrounds to control for additional stimuli variability and enable definitive conclusions on the true effect of background on first impressions of trustworthiness and competence in videoconferencing.

It is also possible that our gender effect was driven by the higher proportion of females in the sample. It has been shown that females tend to judge trustworthy faces as significantly more trustworthy than males (but no differences were found in untrustworthy or neutral faces) [91]. Here we have a female to male participant ratio of approximately 2:1, so perhaps future studies could work to address this.

Despite a convincing effect of video background, facial expression, and gender on first impressions in videoconferencing, the generalisability of the results cross-culturally remains to be examined. The sample consisted of western participants which could be problematic as culture influences contextual processing. Research indicates that East Asian individuals attend more to contextual factors than Western individuals [92, 93]. If the study was conducted in an eastern sample, then different backgrounds may influence trait evaluations differentially This is particularly pertinent as videoconferencing is used for global connections, with virtual meetings used to reduce travel. It is therefore likely that in first virtual interactions individuals may be connecting with people around the world. More research is subsequentially needed to establish cultural differences in the perception of virtual backgrounds the findings are generalised and implemented into recommendations.

### Implications and conclusions

The COVID-19 pandemic induced a global boom in videoconferencing which has quickly developed into a permanent feature of the post-pandemic world. In the professional environment, 75% of business meetings are predicted to occur by videoconferencing by 2024 [23]. The findings of this study therefore have extensive implications for professional organisations and the general public.

As the use of virtual interviews increases, the findings are highly relevant to recruitment processes because competence is a strong predictor of hire ability [94]. Candidates should therefore be aware of their backgrounds to make a good first impression. Beyond the boardroom, the implications of the study are pervasive for the criminal justice as defendants are increasingly appearing by videoconferencing [95]. Based on this study’s results, it is feasible that video background will influence perceptions of defendants, particularly as Blair [14] demonstrated influence of appearance on sentencing and judicial decisions.

For those regularly using videoconferencing platforms such as Zoom and Teams, the study implies a recommendation for a background with a bookcase or house plants. Novelty virtual backgrounds and showing your full living space should be avoided. Males in particular should be aware of their backgrounds. Individuals are also recommended to smile to elicit the best first impressions of trustworthiness and competence and negate the negative effects of background, particularly for those who have no choice but to have their home in view.

The present study has demonstrated the influence of video background, facial expression, and gender on first impressions of trustworthiness and competence in a videoconferencing context. Further, it found interaction effects between background and both facial expression and gender. These findings have implications for those who regularly use videoconferencing as video background affects trait evaluations, particularly as videoconferencing develops into a permanent feature of the professional environment. Overall, the current study demonstrates the power and influence of video background on first impressions, as well as the importance of being able to negate these influences in order to leave the best first impression possible.
